# On-Board Detection of Pedestrian Intentions

**DOI:** 10.3390/s17102193

**Published:** 2017-09-23

**Authors:** Zhijie Fang, David Vázquez, Antonio M. López

**Affiliations:** 1 Computer Science Department, Universitat Autònoma Barcelona (UAB), 08193 Barcelona, Spain; 2 Computer Vision Center (CVC), Universitat Autònoma Barcelona (UAB), 08193 Barcelona, Spain; dvazquez@cvc.uab.es (D.V.); antonio@cvc.uab.es (A.M.L.)

**Keywords:** pedestrian intention, ADAS, self-driving

## Abstract

Avoiding vehicle-to-pedestrian crashes is a critical requirement for nowadays advanced driver assistant systems (ADAS) and future self-driving vehicles. Accordingly, detecting pedestrians from raw sensor data has a history of more than 15 years of research, with vision playing a central role. During the last years, deep learning has boosted the accuracy of image-based pedestrian detectors. However, detection is just the first step towards answering the core question, namely *is the vehicle going to crash with a pedestrian provided preventive actions are not taken?* Therefore, knowing as soon as possible if a detected pedestrian has the intention of crossing the road ahead of the vehicle is essential for performing safe and comfortable maneuvers that prevent a crash. However, compared to pedestrian detection, there is relatively little literature on detecting pedestrian intentions. This paper aims to contribute along this line by presenting a new vision-based approach which analyzes the pose of a pedestrian along several frames to determine if he or she is going to enter the road or not. We present experiments showing 750 ms of anticipation for pedestrians crossing the road, which at a typical urban driving speed of 50 km/h can provide 15 additional meters (compared to a pure pedestrian detector) for vehicle automatic reactions or to warn the driver. Moreover, in contrast with state-of-the-art methods, our approach is monocular, neither requiring stereo nor optical flow information.

## 1. Introduction

**Figure 1:**
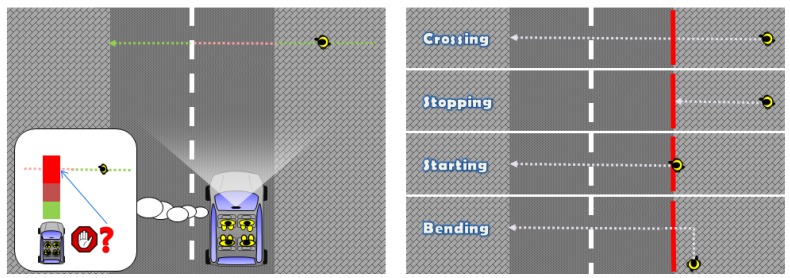
(**Left**) anticipating as much as possible the intentions of a pedestrian allows for safer and more comfortable maneuvers. For instance, we would like to know if the pedestrian is going to enter the road while walking towards it from the sidewalk; or, in general, if it is going to enter a critical area that the ego-vehicle can compute as its predicted driving path; (**Right**) different situations taking the curbside (red line) as Reference [12]. From top to bottom: a pedestrian will be *crossing* the road without *stopping*; a pedestrian walking towards the road will be *stopping* at the curbside; a pedestrian that was stopped at the curbside is *starting* to walk for entering the road; a pedestrian walking parallel to the curbside (parallel to the trajectory of the ego-vehicle) will be *bending* towards the road. Here, we plot the pedestrian walking away from the ego-vehicle, but walking towards the ego-vehicle and *bending* would fall in the same category.

Avoiding vehicle-to-pedestrian crashes is a critical requirement for nowadays advanced driver assistant systems (ADAS) and future self-driving vehicles. Accordingly, detecting pedestrians from raw sensor data has a history of more than 15 years of research, with vision playing a central role [1]. During the last years, deep learning has boosted the accuracy of image-based pedestrian detectors [2]. However, detecting the pedestrians is just an intermediate step since the question to answer is if the ego-vehicle is going to crash with a pedestrian provided preventive actions are not taken. For instance, using Figure 1 Left as reference, a pure pedestrian detection approach would report that a pedestrian may be in danger as a function of his or her location with respect to the road ahead of the ego-vehicle, his or her distance to the vehicle, and the vehicle motion (direction and speed). However, knowing as soon as possible if a detected pedestrian has the intention of intersecting the ego-vehicle path (expecting the vehicle slowing down or braking) is essential for performing safe and comfortable maneuvers preventing a crash, as well as having vehicles showing a more respectful behavior with pedestrians (see Reference [3]).

Despite the relevance of detecting pedestrian intentions, since pedestrian detection is the first hard task to solve, most of existing literature focuses on the latter topic as can be seen in the surveys [1,4,5,6], and relatively little on the former one [7,8,9,10,11,12,13,14,15,16,17]. This paper aims at contributing in this line by presenting a new vision-based approach that analyzes the pose of a pedestrian along several frames to determine if he or she is going to enter a road area that may generate a risk of crashing. The presented method relies on: (a) a CNN-based (Convolutional Neural Network) pose estimation method that detects pedestrians and provides their skeleton simultaneously [18]; (b) a fast classifier based on a set of high-level features extracted from a detected skeleton and a normalized SVM (Support Vector Machine) that processes them. The literature of action recognition in videos. supports the hypothesis that high-level features (e.g., skeleton joints) are more action-informative than low-level ones (e.g., HOG, HOF) [19]. In addition, since the pose estimation method is a single-frame monocular approach, in contrast with state-of-the-art methods for detecting pedestrian intentions, ours neither requires stereo nor optical flow information.

For the present study, we rely on a publicly available dataset designed to assess methods for detecting pedestrian intentions [7]. In this dataset, it is considered that a pedestrian enters in a risk area when he/she moves from the sidewalk towards the road ahead of the ego-vehicle, as seen in Figure 1 Right. We present experiments showing 750 ms of anticipation for pedestrians crossing the road, which, at a typical urban driving speed of 50 Km/h, can provide 15 additional meters (compared to a pure pedestrian detector) for vehicle automatic reactions or to warn the driver. At the same speed, initiating emergency brake with 160 ms of anticipation over a 660 ms time to collision can reduce the chance of injury requiring hospitalization from 50% to 35% [20].

The rest of the paper is organized as follows. In Section 2, we summarize the works most related to this paper. In Section 3, we describe our approach for detecting pedestrian intentions. In Section 4, we present the performed experiments and discuss the obtained results. Finally, Section 5 draws the conclusions and future work.

## 2. Related Work

One of the first attempts of predicting pedestrian future is more related to pedestrian path prediction, i.e., without an explicit step for determining the intentions of the pedestrians [7]. Pedestrian dynamic models are proposed conveying location, speed and acceleration. The measurements to set such variables come from an HOG/Linear-SVM based pedestrian detector [21] operating on dense stereo images at 16 fps. An Interacting Multiple Model based on Kalman Filters (IMM-KF) is used to predict the future path (<2 s) of a pedestrian according to the used dynamic model and vehicle ego-motion compensation. Overall, a simple constant speed velocity model (with white noise acceleration) was on par with more sophisticated models. In a following work [9], results are improved by considering Gaussian process dynamical models and a probabilistic hierarchical trajectory matching (involving particle filters, PCA and mean-shift). In this case, not only stereo data is used, but the dynamical models also rely on motion features extracted from dense optical flow with vehicle ego-motion compensation. Intuitively, the method implicitly tries to predict how the silhouette of a tracked pedestrian evolves over time. Moreover, it explicitly assessed the question of whether a pedestrian will cross from the side walk to the road ahead of the ego-vehicle, i.e., *crossing* vs. *stopping* in Figure 1 Right. For doing that, trajectories of the *stopping* and *crossing* classes are learned and, then, unobserved testing trajectories are classified according to the trajectory matching method.

In this paper, we present an explicit data-driven model to detect pedestrian intentions using skeleton features, which are used without requiring to individually track them. In fact, tracking is only assumed for a pedestrian as a whole, which is unavoidable for any method aiming at detecting intentions. Our proposal obtains equivalent results to [9] in the *crossing* vs. *stopping* classification, being much simpler and only relying on monocular information, neither on dense stereo as in [7,9], nor on dense optical flow with ego-motion compensation as in [9].

In Reference [11], a stereo-vision system is also used to assess the silhouette of the pedestrians for determining their intentions (other authors used $360^{\circ }$ LIDAR [15]). The proposed method has the advantage over previous ones of requiring vehicle ego-motion compensation only for tracking of the pedestrians, but not for computing features for detecting intentions. It is argued that such a compensation would need to be too precise to preserve small pedestrian movements (i.e., more precise than for tracking), which are crucial for recognizing intentions. As in [11], the method that we present here does not require ego-motion compensation by itself (only if the tracking uses it). Moreover, our results are comparable (in fact, slightly better) to [11] without requiring dense stereo.

Other approaches focus on on-board head and body orientation estimation as a cue for detecting the intention of a pedestrian, from monocular [10] or stereo [12,13] images with vehicle ego-motion compensation. However, it is unclear how we actually can use these orientations to provide intention estimation, neither how much additional time this information can bring to perform a reactive maneuver. Indeed, for a time to collision below 2 s, pedestrians tend to look at the vehicle before crossing [17]. However, we are not aware of any work reporting with how much anticipation this happens; for instance, in [17], pedestrian behavior statistics are based on observations at the point of crossing (e.g., the curbside in Figure 1 Right). In our proposal, we rely on a 2D pedestrian pose estimation method; therefore, we are already implicitly taking into account the kind of body orientation that works such as [13] try to compute; in fact, the one we use is more fine grained. The method used to obtain the pose also provides head orientation; however, it is not as robustly detected as the rest of the body. Thus, we consider head pose estimation as an additional cue we could consider in the future since it can complement our current study. On the other hand, the experiments reported in [12] suggest that head detection is not useful for distinguishing *crossing* vs. *stopping*, although it is for detecting *bending*.

In Reference [17], it is suggested to further study the gait patterns of pedestrians, which is what our method actually do by using a data-driven approach. In fact, in [14], it is explicitly said that a lack of information about the pedestrian’s posture and body movement results in a delayed detection of the pedestrians changing their crossing intention. Thus, our proposal of using a 2D pose estimator for analyzing intentions is aligned with these suggestions.

## 3. Detecting Pedestrian Intentions

### 3.1. Our Proposal in a Nutshell

**Figure 2:**
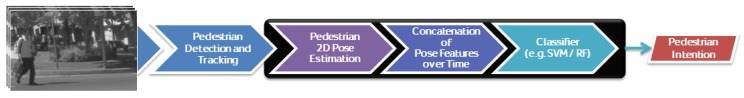
Proposed method. Monocular frames are continuously acquired and processed for detecting and tracking pedestrians. For each tracked pedestrian, our proposal consists of: estimating his or her 2D pose by skeleton fitting, computing features from the fitted skeleton; input them to a learned classifier, which will output the intention of the pedestrian.

**Figure 3:**
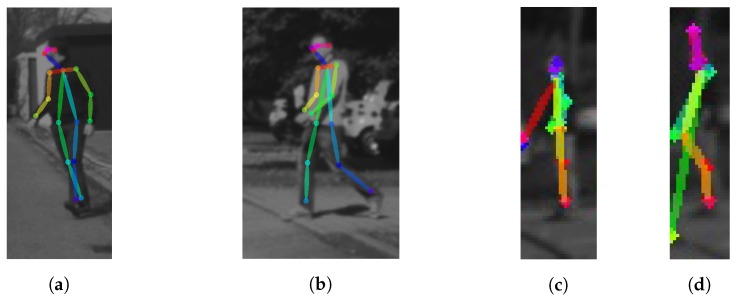
2D pose estimation, i.e., 2D skeleton fitting, at increasing pedestrian-vehicle distances. (**a**) 13 m; (**b**) 18 m; (**c**) 40 m; (**d**) 45 m.

**Figure 4:**
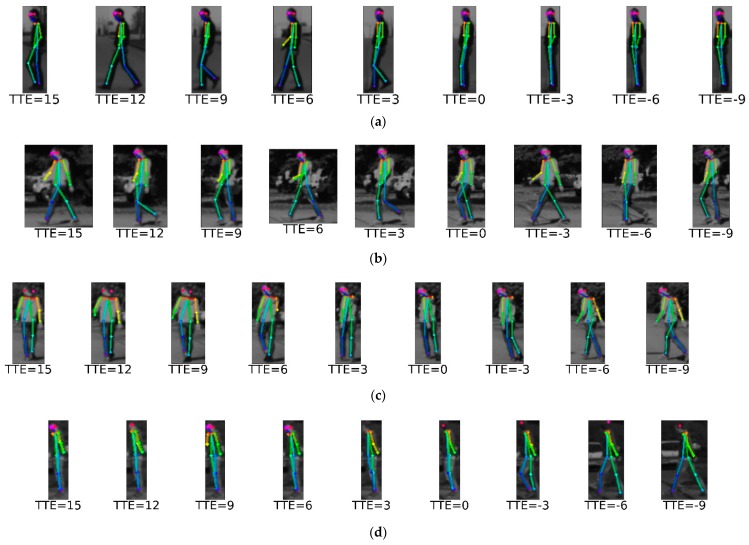
Skeleton fitting for the four situations considered in this paper. We show a sequence for each situation. TTE stands for time to event. TTE = 0 is when the event of interest happens: *stopping* at the curbside, crossing the curbside, *bending*, and starting to walk from the curbside. Positive TTE values correspond to frames before the event, negative values to frames after the event. (**a**) *stopping*; (**b**) *crossing*; (**c**) *bending*; (**d**) *starting*.

The proposed approach is summarized in Figure 2. The first step consists of pedestrian detection and tracking, which is a common step to any method assessing pedestrian intentions. We are agnostic to the methods used for these tasks, we only assume that, for each pedestrian, we will have a 2D bounding box (BB) that comes from the combination of detection and tracking. The second step consists of the use of a 2D pose estimation method that results on the fitting of a skeleton model to the pedestrian contained in each BB. In this case, we propose the use of the recent method presented in [18]. It relies on a two-branch multi-stage CNN trained on the Microsoft COCO 2016 keypoints challenge dataset [22]. When applied to a BB containing a pedestrian, it is able to perform the skeleton fitting being robust to pedestrian shifts (because inaccuracies in the detection and tracking step) and scaling (because different pedestrian sizes and distance to the camera) within the BB. Figure 3 shows different skeleton fittings as a function of the distance. The algorithm starts to fail only at large distances (e.g., 40 m in the figure’s example). The third step consists of extracting a feature vector, namely ψ, based on the skeleton fitted to each tracked pedestrian (Section 3.2). In fact, since intentions are shown as an action over time, for each tracked pedestrian, at frame *t*, we concatenate the feature vectors of the last *T* frames, giving rise to a per-pedestrian feature vector $\Psi_{t}=<\psi_{t},\psi_{t-1},\ldots ,\psi_{t-T}>$, where $\psi_{i}$ stands for the feature vector at frame *i*. Figure 4 shows skeleton fitting results for BBs coming from 10 consecutive frames (T=10) depicting pedestrians performing the four situations we are considering in this paper. The final step consists of applying a classifier C on Ψ that fires for a pedestrian intention we want to assess (Section 3.3).

Note that the proposed method does not explicitly require global egomotion compensation. The detection-tracking process is already sufficient to capture the pose evolution on which our method relies. Therefore, explicit egomotion compensation would be required only if the tracking itself relies on it.

### 3.2. Skeleton Features

**Figure 5:**
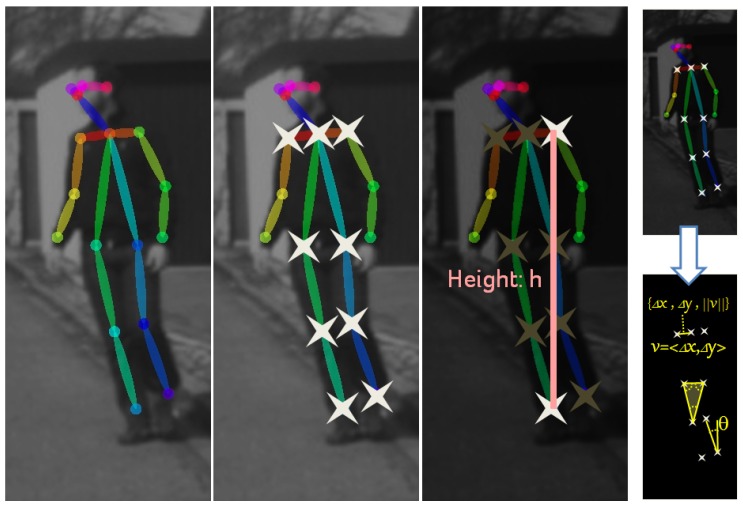
Skeleton fitting is based on 18 keypoints, distinguishing left and right arms and legs [18]. We use the nine keypoints highlighted with stars. The upper keypoint among those and the lower are used to compute height *h*, which is used as scaling factor for normalizing the keypoint coordinates. Then, using the normalized keypoints, different features based on relative angles and distances are computed as features. For instance, to the right, we see several examples: (1) distance in the *x* (column) and *y* (row) axes and Euclidean distance between two keypoints (Δx, Δy, ∥v∥); (2) angle between two keypoints (θ); (3) the three angles of a triangle formed by three keypoints. After normalizing by *h* these seven values, they become components of the feature vector $\psi_{i}$ of frame *i*. Computing similar values by taking into account all the keypoints we complete $\psi_{i}$.

In Figure 5, we can see that the fitted skeleton is based on 18 keypoints. Note that left and right body parts are distinguished. However, not all keypoints are always located very accurately when processing on-board images. We found as most stable the nine keypoints highlighted with a star, which correspond to the legs and to the shoulders. Note that these are highly relevant keypoints since ultimately the legs are executing the pedestrian intentions of continue/start walking or stopping; while having keypoints from shoulders and legs provides information about global body orientation.

From the selected keypoints, we compute features. First, we perform a normalization of keypoint coordinates according to a factor *h* defined as shown in Figure 5, which is proportional to the pedestrian height. Then, different features (conveying redundant information) are computed by considering distances and relative angles between pairs of keypoints, as well as triangle angles induced by triplets of keypoints. In total, we obtain 396 features (dimension of ψ). Since we concatenate the features collected during the las *T* frames, our feature vector Ψ has dimension 396T.

It is worth mentioning that we know the position of any keypoint along the different frames because they correspond to an specific and unique anatomical part of the fitted skeleton. Thus, a priori, it makes sense to account for keypoint time differences. In fact, we did it; however, results did not improve and thus we discarded across-frame features. We think the reason is that the proposed Ψ already conveys sufficient information to perform the further classification task.

### 3.3. Classifier

In this paper, we consider binary classifiers that rely on learned frontiers and output a normalized score. In particular, we tested the Random Forest (RF) and Support Vector Machine (SVM) methods. RF is able to learn nonlinear frontiers and outputs a probability value. For the SVM, we apply Platt scaling on RBF (Radial Basis Function) Kernel scores. We access all these functionalities by using scikit-learn [23].

Independently of using SVM or RF, following the literature evaluation protocols [11,12], in this paper, we assume a procedure for detecting pedestrian intentions that is based on the following binary classifiers:

- $C_{c}$: Continue walking perpendicularly to the camera (∼*crossing*) vs. *stopping*.
- $C_{b}$: Continue walking parallel to the camera vs. *bending*.
- $C_{s}$: Continue stopped vs. starting to walk perpendicular to the camera.

Note that *Continue walking perpendicularly to the camera* is equivalent to *crossing* given a fiducial point of interest such a curbside or a frontier of risk determined by the ego-vehicle future motion.

Each classifier can have a threshold to determine if it fires or not. With a simple pedestrian tracking, we may need to test all classifiers, while, with a tracker that keeps proper pedestrian motion vectors, we may need to apply only one of those classifiers.

## 4. Experimental Results

### 4.1. Dataset

Unfortunately, at the moment of doing this research, the only publicly available dataset (to the best of our knowledge) with ground truth (GT) annotations for assessing pedestrian intentions is the one first introduced in [7] and recently used in [11,12]. The dataset contains 68 sequences (9135 frames in total) recorded on-board with a stereo camera (here, we only use the left frame of each pair) placed in the windshield forward facing the road ahead. The images are taken at 16 FPS (Frames per Second) and their resolution is of 1176×640 pixels. Among the sequences, 55 were taken with vehicle speeds ranging from 20 to 30 km/h, while, for 13, the vehicle was standing. In order to make easier comparisons, the sequences are separated into training and testing as can be seen in Table 1. The pedestrians come with two types of BBs, namely manually provided GT BBs and BBs from a HOG/Linear-SVM classifier. Event tags are provided (*crossing*, *stopping*, *bending*, *starting*) as well as the time to event (TTE) in frames (Figure 4).

### 4.2. Evaluation Protocol

Since we consider the same set of intentions as [11], we also use the same train–test partition of the working sequences (shown in Table 1). We also follow the recommendation of [11] to select positive and negative samples when training the classifiers; i.e., we divide a training sequence in three segments of samples: positives—not used—negatives. We will use the notation A-B, with A>B, meaning that frames with TTE>A are used as positive samples, and frames with TTE≤B are used as negative samples; thus, frames with TTE∈(B,A] are ignored during training.

As in [11,12], we use plots of intention probability vs. TTE. With this type of plot, it is easy to see how many frames we can anticipate a pedestrian action (e.g., for *crossing* vs. *stopping*), or how fast we can react to it (e.g., for starting and *bending*). Since there are several testing sequences per intention, mean and standard deviation are plotted. In addition, also following [11], we use these plots to select a proper probability threshold so that we can also present plots of what they call accuracy vs. TTE. However, we prefer to call it predictability, i.e., for each TTE, a normalized measurement is given of how feasible it is to detect the action under consideration at that TTE. This predictability measurement is computed as follows. First, since the testing sequences have different lengths, we align them by making their TTE = 0 frame to coincide. Then, from the minimum TTE over all the sequences until the maximum TTE, we compute a predictability value for each TTE as follows. All the frames corresponding to the current TTE (i.e., coming from the different testing sequences) are considered. For each of those frames, we apply our method given a classification threshold for the probability of the intention/action under consideration. Then, we divide the number of frames rightly classified by the number of total frames evaluated. Predictability zero indicates that we cannot detect the intention/action, while predictability one means that we can.

Again, following [11,12], we use both the GT pedestrian BBs as well as the detections provided by the HOG/Linear-SVM. Although human-provided BBs are not necessarily consistent, we can take them as the output of a state-of-the-art pedestrian detection and tracking system (nowadays, it could rely on CNN-based models). The hyper-parameters of the classifiers are set here as the ones providing the best performance. For the SVM classifier, C was adjusted by starting in 1 and applying a factor of ×10 until $10^{6}$. C = 10,000 provided the best results. Small variations around this value did not provide significant better results. For the RF, we tested different depths ranging from 7 to 29 in steps of 2, and using 100, 200, 300, and 400 trees. Finally, we selected 21 as depth and 300 trees. The HOG/Linear-SVM classifier is nowadays far from the state-of-the-art, but we use it for a proper comparison with [11,12] in terms of pedestrian intentions. However, we have not implemented a tracker for extrapolating detections from previous frames to a frame where a pedestrian is missed by the HOG/Linear-SVM detector, the reason is that we have quantified these cases as ≈9%; thus, when this happens, we take the corresponding GT BB and add a 10% noise to its defining coordinates (this noise level is used also in [9,12] for perturbing GT BBs).

We have not worked on code optimization; thus, we are not including an in deep analysis of computation time. However, we can indicate several reference times. At testing time, the pose estimation method runs at 10 frames per second in a consumer graded GPU (NVIDIA GeForce GTX-1080, (NVIDIA, Santa Clara, CA, USA)) [18]. Our non-optimized code, which uses estimated poses to predict pedestrian intentions, takes less than 15 ms in an INTEL Xeon E5-1620 v3 PC ( INTEL, Santa Clara, CA, USA). Thus, the main computation time corresponds to pose estimation. In training time, given the already trained pose estimation CNN model, each of our classifiers for detecting pedestrian intentions is trained in approximately one hour.

### 4.3. Crossing vs. Stopping

**Figure 6:**
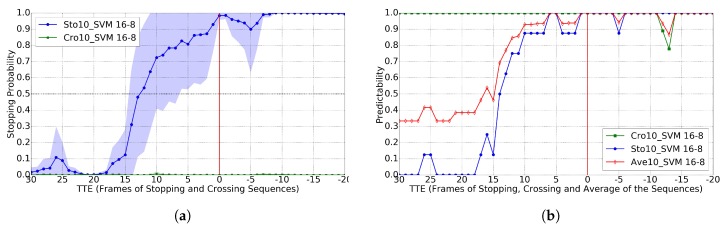
Results for the *crossing* vs. *stopping* classification task ($C_{c}$), using GT (ground truth) pedestrian BBs (Bounding Box), a time sliding window of 10, the RBF-SVM classifier and 16–8 as a trade-off for setting positive and negative frames during training. ’Cro’ curve means applied to testing sequences of crossing, ’Sto’ curve means applied to testing sequences of stopping. Note that the frames from the stopping sequences are rightly classified if $C_{c}>0.20$, while for the crossing sequences those are the wrongly classified. (**a**) classification probability (mean as curves, standard deviation as colored areas); (**b**) predictability for $C_{c}$ with threshold 0.20.

**Figure 7:**
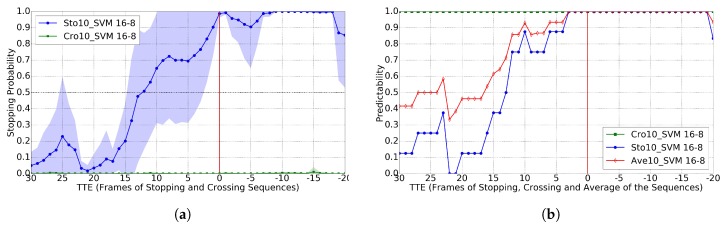
Analogous to Figure 6, but using the BBs of the provided pedestrian detections. (**a**) classification probability; (**b**) predictability for $C_{c}>0.20$.

In the sequences of the used dataset, we can see that the walking cycle is of ≈10 frames; therefore, for developing $C_{c}$ (Crossing vs. Stopping), we started with a temporal sliding window of T=10 as well as using an RBF-SVM frontier. We also set the best performing A-B pair in [11], i.e., 16-8. Figure 6 shows the results of comparing the probabilities of *crossing* vs. *stopping* for different TTE values, as well as the accuracy for a selected threshold; this case corresponds to the use of GT pedestrian BBs. Figure 6a shows that, when we apply $C_{c}$ to the crossing sequences, the probability values are almost zero with very low standard deviation; while, when applied to stopping sequences, the probability starts to grow significantly in the TTE range of 15–10 (in these sequences, TTE = 16 corresponds to one second of anticipation). Thus, the classifier is very sure about when to stop, which is very important from the point of view of safety. By setting a probability threshold of 0.2, we can see in Figure 6b that at TTE = 12 we reach the 0.8 of average predictability. Note that TTE = 12 are 750 ms before the event, which is very interesting, since, in [9], it is reported that humans reach 0.8 predictability with less anticipation, namely 570 ms. Thus, although a comprehensive human-vs.-machine comparison is out of the scope of this paper, this evidence suggests that our prediction system may be on pair with humans for this task. Moreover, in Figure 7, we can see that, when using the BBs of a basic pedestrian detector (HOG/Linear-SVM hear), the results are very similar, also with TTE = 12 for the 0.8 of predictability.

**Figure 8:**
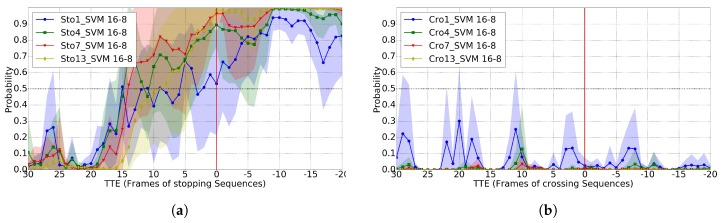
Classification probability for several temporal sliding windows (T∈{1,4,7}) applied to stopping and crossing sequences. (**a**) stopping sequences; (**b**) crossing sequences.

For the GT BBs case, Ref. [11] reports TTE = 11 for the 0.8 of predictability, so our results are comparable but not requiring dense stereo. For the BBs coming from pedestrian detection, Ref. [11] reports TTE = 8 for the 0.8 of predictability, while our method still reports TTE = 12. We think this is due to the fact that our proposal relies on higher level features (based on skeleton keypoints), an observation also reported on action recognition in videos [19]. Moreover, the used 2D pose estimation methods add shift invariance to the exact pedestrian location within the detection BBs, which use to come with inaccuracies. In addition, although it is difficult to report a direct comparison with [12] because accuracy is not reported, looking at the plot of stopping probability vs. TTE for stopping scenarios, it seems that the method proposed in [12] is not robust; in fact, the authors themselves report that head detection is not useful for this particular task (while it is for *bending* actions). In order to complement our study, we also checked the results when using different sizes of temporal sliding window; in particular, we also tested T∈{1,4,7,13}. Results can be seen in Figure 8 when using GT BBs. Note how results improve as we increase *T*; however, these results are not as good as when using T=10 as seen by comparison with Figure 6. When using BBs coming from the HOG/Linear-SVM pedestrian detector, the results are analogous; thus, we do not plot them here for the sake of simplicity.

For these experiments, we also used the RF method; however, for achieving the 0.8 of predictability, we have TTE = 6, which is significantly worse. Obviously, this does not imply that RBF-SVM is better than RF in general, and we only report the result that we obtained for this task given the available training and testing sets.

### 4.4. Bending

Following [11], for training $C_{b}$, we set the A-B pair as 4-0. Again, we report only results for T=10 and RBF-SVM since for T∈{1,4,7} and RF they were worse. In this case, we would like to mention that rather than predicting the intention of *bending*, which is extremely difficult, the aim is to understand that this is happening as soon as possible.

**Figure 9:**
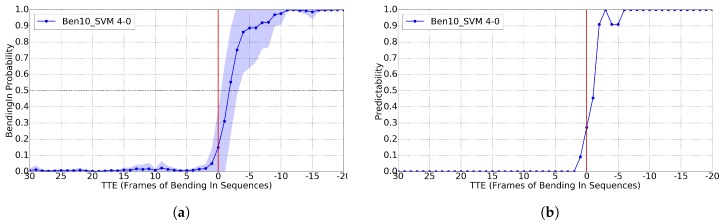
Results for the *bending* classification task ($C_{b}$), using GT pedestrian BBs, a time sliding window of 10, the RBF-SVM classifier and 4-0 as trade off for setting positive and negative frames during training. ’Ben’ curve means applied to testing *bending* sequences. (**a**) classification probability; (**b**) predictability for $C_{b}>0.16$.

**Figure 10:**
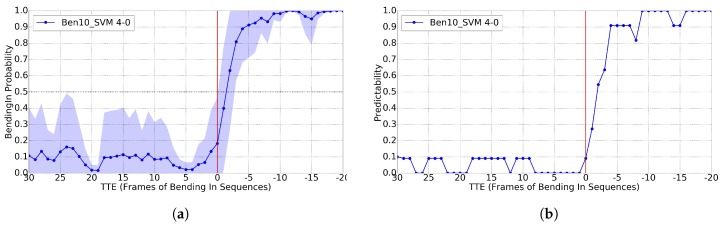
Analogous to Figure 9, but using the BBs of the provided pedestrian detections. (**a**) classification probability; (**b**) predictability for $C_{b}>0.80$.

In Figure 9, we can see that, for GT BBs, we reach the 0.8 of predictability for TTE = -2, i.e., after 125 ms of the event happening. In Figure 10, we plot the analogous results using the BBs from the pedestrian detector. We see that, before the action happens, the system outputs less stable probabilities. However, by using the proper threshold, we still reach 0.8 predictability for TTE = -4 (250 ms). Note that [11] reports TTE = -4 when using GT BBs, and TTE = -5 for BBs from pedestrian detections (312 ms).

We have visually inspected the result and found that for far pedestrians (TTE > 10 since the vehicle is approaching the pedestrian in this case), the 2D pose estimation has difficulties in distinguishing back and front pedestrian views, which introduces an instability that induces differences in training an testing time. This is why in Figure 10a the probabilities fluctuate more for TTE > 10. On the other hand, comparing to Figure 9a, it seems that, at far distances, by just having a more accurate pedestrian detector and thus providing more accurate BBs, can already help the pose estimator. In any case, this back/front viewpoint confusion is a point for improvement in our future work. We think that, for this particular action, head orientation can be also tested to assess if we can predict the action more closely to TTE = 0.

### 4.5. Starting

As can be seen in Table 1, there are too few sequences of this type. Therefore, we have augmented the training set with frames coming from the training sequences of *crossing*, *stopping* and *bending*. In particular, frames from the crossing sequence are taken as positive samples of starting, as well as frames from *bending* sequences with TTE < 0 and stopping sequences with TTE > 4; i.e., all the cases when we see the pedestrians in side view walking. As negative samples, we have taken frames from *stopping* sequences with TTE < 0 and from bending sequences with TTE > 4; i.e., when the pedestrians are not in side view walking. At this point, we would like to comment that we tried also analogous training data augmentation for the previous classifiers ($C_{c},C_{b}$), but results were more noisy, so we have not reported them here for the sake of simplicity.

**Figure 11:**
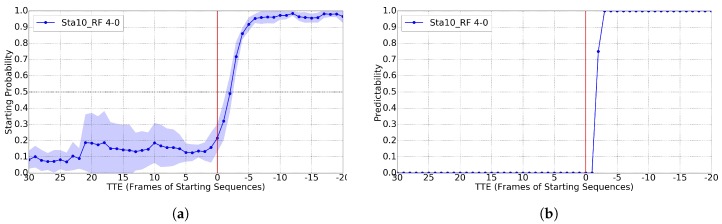
Results for the starting classification task ($C_{s}$), using GT pedestrian BBs, a time sliding window of 10, the RF classifier and 4-0 as trade off for setting positive and negative frames during training. ’Sta’ curve means applied to testing starting sequences. (**a**) classification probability; (**b**) predictability for $C_{s}>0.50$.

**Figure 12:**
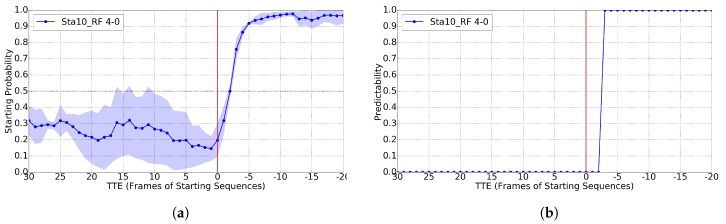
Analogous to Figure 11, but using the BBs of the provided pedestrian detections. (**a**) classification probability; (**b**) predictability for $C_{s}>0.60$.

As for starting, it is rather difficult to predict the action before it happens, the aim is to understand that it is happening as soon as possible. Following [11], for training $C_{s}$, we set the A-B pair as 4-0. In this case, we report results for T=10 and RF, since they are better than for RBF-SVM; however, again, values of T∈{1,4,7} provide worse results. Figure 11 shows the case for GT BBs and Figure 12 for BBs from pedestrian detection. In both cases, we see a predictability of over 0.8 already for TTE = 3 (187 ms). [11] reports TTE = 4 (250 ms). For TTE > 0, Figure 12a shows worse results than Figure 11a due to similar reasons than in bending, i.e., pedestrians are further away and the detection works worse, and this may have impact on the pose estimation if the detection BBs is too noisy.

## 5. Conclusions

The state-of-the-art on-board detection of pedestrian intentions is not so extensive, especially compared to pedestrian detection and tracking. The proposed methods rely on dense stereo data and/or dense optical flow. In this paper, we have shown how modern CNN-based off-the-shelf 2D pedestrian pose estimation methods can be used to develop a detector of pedestrian intentions from monocular images. On top of a fitted human skeleton, we have defined keypoint relative features, which, together with well grounded and efficient machine learning methods (SVM, RF), allowed us to address the detection of situations such as *crossing* vs. *stopping*, *bending*, and *starting*. We showed that feature concatenation over a time sliding window of ten frames gives rise to results that are even better than the state of the art based on processing dense stereo data. Our experiments show anticipation of 750 ms regarding a pedestrian that will cross the road, 250 ms after a pedestrian performs a bending action, and 187 ms when a pedestrian starts to enter the road after being on a standstill state. There are still difficult cases, especially when the pedestrians are seen in back or frontal views at far distance, since then the pose estimation can fluctuate in the skeleton adjustment (confusing left and right body parts). This affects bending detection; thus, it will be one of our first addressed future works. In addition, interesting future work consists of assessing the same pedestrian intention scenarios when there are more pedestrians, eventually occluding each other, which must start by producing a proper dataset with such cases.

## Figures and Tables

**Table 1:** Number of sequences of training and testing for each type of pedestrian intention [7].

	Stopping	Crossing	Bending	Starting
Training	9	9	12	5
Testing	8	9	11	4
Total	17	18	23	9
Vehicle Moving	12	15	18	9
Vehicle Standing	5	3	5	0
